# Structure and Morphological Properties of Cobalt-Oxide-Based (Co_3_O_4_) Materials as Electrodes for Supercapacitors: A Brief Review

**DOI:** 10.3390/ma18020413

**Published:** 2025-01-17

**Authors:** Maxwell F. L. Garcia, Luis C. C. Arzuza, Gelmires A. Neves, Francisco J. A. Loureiro, Marco A. Morales, Daniel A. Macedo, Helio L. Lira, Romualdo R. Menezes

**Affiliations:** 1 Laboratory of Materials Technology, Department of Materials Engineering, Federal University of Campina Grande, Campina Grande 58400-850, Brazil; luisarzuza179@gmail.com (L.C.C.A.); gelmires.neves@ufcg.edu.br (G.A.N.); helio.lucena@professor.ufcg.edu.br (H.L.L.); 2 TEMA—Centre for Mechanical Technology and Automation, Department of Mechanical Engineering, University of Aveiro, 3810-193 Aveiro, Portugal; francisco.loureiro@ua.pt; 3 LASI—Intelligent Systems Associate Laboratory, 4800-058 Guimarães, Portugal; 4 Department of Theorical and Experimental Physics, Federal University of Rio Grande do Norte, Natal 59078-970, Brazil; marco.moralestorres@gmail.com; 5 Materials Science and Engineering Postgraduate Program, Federal University of Paraiba, João Pessoa 58051-900, Brazil; damaced@gmail.com

**Keywords:** supercapacitors, cobalt-based materials, Co_3_O_4_ electrodes, energy storage devices

## Abstract

Over the past 15 years, there has been a significant increase in the search for environmentally friendly energy sources, and transition-metal-based energy storage devices are leading the way in these new technologies. Supercapacitors are attractive in this regard due to their superior energy storage capabilities. Electrode materials, which are crucial components of supercapacitors, such as cobalt-oxide-based electrodes, have great qualities for achieving high supercapacitor performance. This brief review presents some basic concepts and recent findings on cobalt-based materials used to fabricate electrodes for supercapacitors. The text also clarifies how morphological characteristics typically influence certain properties. The inner surface of the electrode exhibits several properties that change to provide it a great boost in specific capacitance and charge storage. Porous structures with defined pore sizes and shapes and high surface areas are important features for improving electrochemical properties. Finally, we present some perspectives for the development of cobalt-oxide-based supercapacitors, focusing on their structure and properties.

## 1. Introduction

In the current scenario, the search for environmentally friendly sources has driven incessant research into new devices that enable balancing the energy demand, which increases every year. This is combined with the imminent future shortage of fossil fuels and the increasing emergence of strategies that reduce the emission of toxic gases into our atmosphere. Energy solutions mainly involve the use of alternative sources, such as wind and solar energy, along with the production of hydrogen and the use of energy storage based on raw materials that are less aggressive to the environment and can thus be used together to achieve an optimal performance [1,2]. In this context, the use of batteries and supercapacitors has been widely studied because they have fast charge/discharge properties and long life cycle stability, in addition to high power density. Lithium-ion batteries, for example, are commonly used as energy stores. However, batteries have a low power density, limiting their use, thus requiring integration from multiple modules. On the other hand, supercapacitors exhibit high power density and a long-lasting lifespan [3,4,5]. The great advantage of supercapacitors is that there are no changes in the phase or chemical composition of their structure during the charge/discharge processes, providing them high cyclic stability and reversibility, and they can be used in a wide temperature range (−40 to 85 °C) [6]. These devices promise to bridge the gap between traditional dielectric capacitors (high output power) and batteries (high energy storage) [7].

In this sense, the literature highlights that transition metal oxides with multiple oxidation states and high capacitance have been widely investigated in electrochemical applications, optical and magnetic materials, gas sensors, photocatalysts, batteries, etc. [8,9,10]. Therefore, the production of materials with structural and morphological characteristics has become a relevant theme of several studies. Electrochemical properties are mainly influenced by dimension, porosity, particle size, and surface characteristics. Such characteristics favor the accessibility of electrolytes and enable a better flow of current during the charging and discharging processes, relating to an important approach to improve electrochemical properties in energy storage applications [11,12,13].

Materials based on oxides of transition metals, such as nickel, iron, manganese, and cobalt (especially Co_3_O_4_), have been reported as typical materials for supercapacitor electrodes. Among these oxides, the interest in cobalt-based materials has increased in studies dealing with supercapacitor electrodes and batteries. Cobalt oxide has electrochemical properties that are favorable to the development of these devices, such as high theoretical specific capacitance (3560 F g^−1^). The high specific capacitance of cobalt oxide is evident compared to other oxides such as RuO_2_ (2200 F g^−1^), MnO_2_ (1370 F g^−1^), NiO (2584 F g^−1^), and V_2_O_5_ (2120 F g^−1^) [14]. In order to overcome the intrinsic problems of cobalt oxides, the addition of small amounts of carbon nanomaterials or transition metals to nanostructured materials can significantly increase the electrochemical properties by accelerating the reaction kinetics, suppressing the aggregation of metal oxide nanostructures, accommodating electrode volume changes, and thus improving the electrode rate and performance [15,16]. Another strategy may be through the integration of two or more metals in a heterogeneous catalyst, inducing the presence of multiple valences of cations with more complex electronic structures and higher electrical conductivity. Therefore, the synthesis of nanostructured materials can achieve better electrode properties from the presence of a significant electroactive surface area and optimal porosity, exhibiting a shorter path for electron and ion diffusion, and balancing the voltages caused by changes in volume during the cycle [17].

This article aims to briefly review and highlight the most interesting and applicable properties, as well as the latest advances in technological applications, of Co_3_O_4_ in electrodes with prominent electrochemical properties. Furthermore, although several studies have been published in recent years, the present focus will prioritize a systematic review focusing on the main interesting and applicable properties and the latest advances in applications of cobalt-oxide-based electrodes as supercapacitors.

## 2. Cobalt-Oxide-Based Electrodes

**Figure 1:**
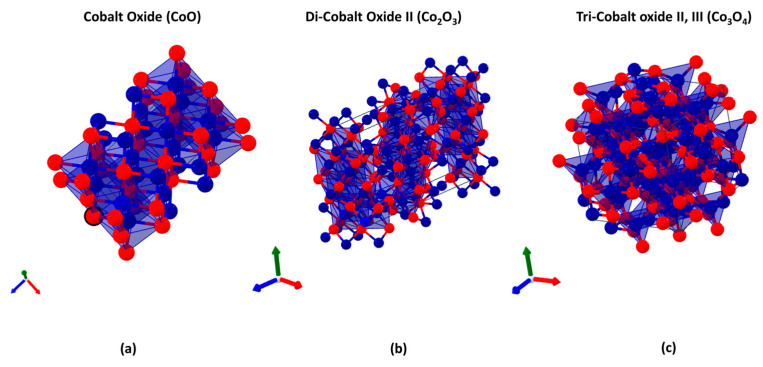
Crystalline structures of Co oxides: (**a**) cobalt oxide; (**b**) di-cobalt II; (**c**) tri-cobalt II, III.

Cobalt oxide (Co_3_O_4_) is the most studied and stable structure for electrochemical applications [18], presenting good stability in corrosive environments and being able to achieve significant values of specific surface area depending on the synthetic route used. Cobalt oxide comes in three different types in nature: cobalt oxide, di-cobalt oxide, and tri-cobalt oxide II, III (Figure 1).

According to the literature, cobalt oxide is stable up to approximately 900 °C in an air atmosphere. Despite this, its performance decreases under extreme thermal conditions, such as high temperatures during operation. In this case, under extreme conditions, the reduction in device performance is due to the acceleration of structural degradation, significant loss of active surface area, and, consequently, evaporation of the electrolyte [19,20]. In this context, strategies to improve thermal stability include doping (e.g., nickel, zinc, or manganese) into the Co_3_O_4_ lattice. This can improve thermal stability and suppress phase changes. Composite material synthesis (combining Co_3_O_4_ with carbon materials, e.g., graphene or carbon nanotubes) can improve thermal conductivity, structural stability, and overall electrochemical performance. Nanostructuring processes (e.g., nanowires, nanotubes, or porous structures) can better tolerate thermal stress due to their high surface area and improved structural flexibility [21,22].

Supercapacitors (see Section 3), electrochemical capacitors, sensors, and batteries commonly use the third type as a base material [23,24,25]. Co_3_O_4_-based electrodes have good redox properties, strong redox capacity, stability of up to 10,000 cycles in alkaline electrolytes, and area capacitance up to 25 F cm^−2^ [26,27]. However, the literature highlights that the specific capacitance achieved by electrodes based on synthetic Co_3_O_4_ is mainly between 200 F g^−1^ and 1000 F g^−1^, values very far from their theoretical specific capacitance, provided as an intrinsic characteristic of the material. This is because cobalt oxide loses its capacitance at high current densities, making it hard to reverse, causing the capacitance to decrease much lower than its theoretical value, which severely limits its efficiency [28]. In this case, making supercapacitor electrodes perform better requires controlling their shape and, using the correct synthesis methods, creating nanostructures that make it easier for ions from the electrolytic solution to enter the electrode matrix. This lowers the electrical resistance between the electrode’s current collector interfaces.

**Figure 2:**
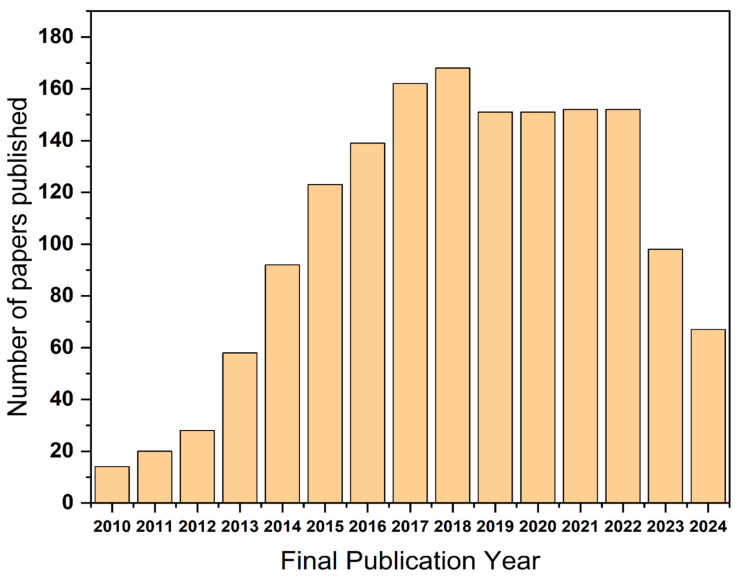
The Web of Science Core Collection contains several papers focused on cobalt-based electrodes for supercapacitors from 2010 to 2024 (keywords used in the search: Co_3_O_4_ and supercapacitor).

In the last 15 years, studies have focused on obtaining Co_3_O_4_ electrodes (see Figure 2) with nanostructural morphologies of 1D, 2D, or 3D, as well as structural modification providing the formation of oxygen vacancies or of the metal itself. Obtaining cobalt oxide nanostructures with different shapes can provide surfaces important properties for processes that convert and store energy. The shape and size of the Co_3_O_4_ nanostructures, the creation of vacancies, stable nanocrystalline phases, and the surface areas of these particles all play major roles in how electrical dynamics occur during charge storage processes [29,30].

More recent approaches apply a modeling approach based mainly on DFT simulation studies using the Synthetic Growth Concept [31,32,33]. These applications are widely used to guide their growth and control morphology during the synthesis of materials of similar complexity, including specific structural and morphological properties.

## 3. Supercapacitors

Supercapacitors are electrochemical devices with high energy storage density and the ability to deliver energy quickly compared to conventional electrolytic capacitors and batteries. Basically, supercapacitors are devices consisting of two porous electrodes (anode and cathode), an electrolyte, and a separator. The electrode primarily facilitates the charge storage process by adsorbing electrolyte ions at the electrolyte/electrode interface.

**Figure 3:**
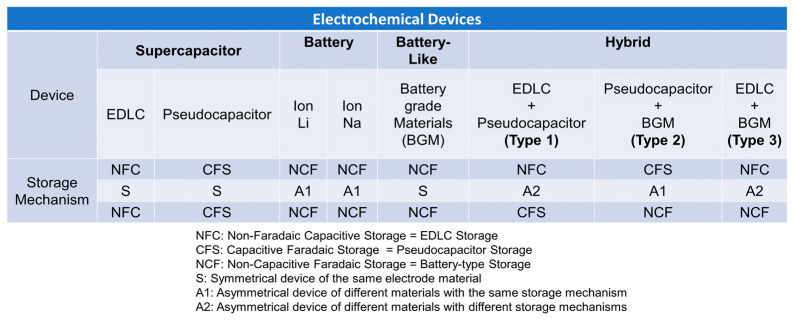
Summary of electrode storage mechanisms for many devices [30].

There are three types of supercapacitors based on how they store charge: electrical double-layer electrodes (EDLCs), pseudocapacitors (redox), and hybrid supercapacitors. Furthermore, a new device called “supercapaterry” (supercapacitor + BGM) [34,35,36] has been proposed to represent devices that take advantage of the capacitive and Faradaic charge storage mechanisms in the material of the electrode. In general, electrostatic interactions accumulate charges on the surface of the electrode, suggesting that increasing the specific surface area improves performance. Note that capacitive charge storage mechanisms can be from non-Faradaic processes (EDLC) or Faradaic processes (pseudocapacitors) or both. On the other hand, Faradaic charge storage can be capacitive (pseudocapacitive) or non-capacitive (battery-like; Figure 3).

Therefore, the combination of these electrodes in the optimization of supercapacitive devices has the same objective, which is to achieve a high power capacity of supercapacitors and a large storage capacity of batteries [37]. Cobaltite oxide has garnered significant attention due to its ability to enhance the storage capacity in supercapacitors by approximately 60% when mixed with graphene electrodes. This is a result of the higher electrical conductivity and increased surface area in one of the electrodes of the supercapacitor [38]. Asymmetric hybrid supercapacitor CoFe_2_O_4_/carbon nanotube (MWCNT)-based electrodes showed superior electrochemical performance when compared with a CoFe_2_O_4_ pure one. The formation of 3D network conductive pathways can enhance the charge and mass transport between the electrodes and electrolytes during faradic redox reactions [39].

### 3.1. Electrical Double-Layer Electrode (EDLC)

The separator, a semi-permeable membrane in the electrical double-layer electrode (EDLC) type, transports ions and prevents the device from short-circuiting. On the other hand, a current collector supports the active material of the supercapacitor electrode, effectively transferring electrons from the active material to external energy sources. The electrode storage mechanisms based on EDLC were developed from the classical models of Helmholtz–Perrin (1908), Stern (1920), and Grahame (1925). This mechanism suggests that the accumulation of charges is defined as the formation of a double electrical layer on the electrode wall as a result of electrostatic attractive forces between the electrolyte ions and the electrode surface [40,41].

The imposed electrical voltage causes an ionic charge layer on the surface, and, in turn, ions of opposite polarity accumulate on the surface of this first ionic layer, forming a second ionic layer. The electrode potential changes as it approaches the diffusion layer, moving from a linear to an exponential regime. As a result, the charge accumulation mechanism of EDLCs achieves high speed during the charge/discharge cycle. Because no chemical reactions occur during the cycle, EDLCs provide a long service life and optimal capacitive characteristics due to the active high specific surface area of the materials [42,43]. Therefore, it is considered to be physical in nature. Carbon materials, such as activated carbon, graphene, and carbon nanotubes, are typically materials used for EDLC electrodes [44,45,46,47].

### 3.2. Redox Pseudocapacitors

Redox pseudocapacitors build up charge through the Faradaic electronic charge transfer reaction on the surface [48]. Because of chemical reactions that occur during fast charge and discharge cycles, reversible redox reactions can result. These reactions can change the structure of the electrode. Materials that are pseudocapacitive have electrochemical properties or a mix of sub-potential deposition, surface redox, and intercalation pseudocapacitance [49]. Sub-potential deposition is when a charge changes as the applied potential changes. Surface redox pseudocapacitance, on the other hand, occurs close to the active material’s surface when charge is captured or relaxed in electrochemically active areas through reversible redox processes involving electron transfer. Redox reactions associated with the intercalation of ions, involving the insertion of ions between the layers of the active material, describe intercalation pseudocapacitance. This phenomenon is not associated with phase transformations or changes in the crystalline structure at the same time that charge transfer processes occur. A well-known example in the literature is a lithium ion that enters the niobium oxide interlayer spaces at high velocity. This occurs because these voids are three times larger than the size of the lithium ion, facilitating this intercalation [50,51]. Therefore, one of the keys to achieving better electrochemical performance is the synthesis of porous electrode materials (mesoporous structures) as this property aids in the diffusion of electrolyte ions during redox charge transfer reactions [50], a fact we will discuss later. Energy storage devices have utilized a variety of materials as electrodes, such as high-electronic-conductivity polymers, metal oxides, and their composites.

The synthesis of supercapacitor electrodes considers the choice in material, which greatly affects the parameters of specific capacitance, charge and discharge cycles, and stability. Researchers have reported methods to enhance the specific capacitances of pseudocapacitor materials, particularly by utilizing nanostructured materials with high surface area/volume ratios in close-to-surface charge storage processes, a beneficial approach for pseudocapacitors and EDLCs. Transition metal oxides (RuO_2_, MnO_x_, CoO_x_, NiO, and Fe_2_O_3_), as well as various composites based on MXenes (based on titanium and carbon), are commonly used materials in pseudocapacitors and battery materials.

### 3.3. Hybrid Supercapacitors

Hybrid supercapacitors attempt to increase their energy density without reducing their power density. These hybrid devices combine the supercapacitor and rechargeable battery characteristics into one device (see the next section). Hybrid supercapacitors consist of different electrochemical electrodes (anode and cathode), which can combine EDLC and pseudocapacitor electrodes, or supercapacitor electrodes with battery electrodes [52]. Thus, hybrid supercapacitors show high energy density, good power density, and a long cycling life. Researchers are focusing on improving these properties to advance the fabrication of these new devices through the specific surface area and porous size distribution of the electrode material (e.g., aqueous zinc-ion [53] and lithium-ion hybrid capacitors [54]). Recently, researchers discovered that the cathode material for lithium-ion hybrid capacitors, edge-rich reduced microcrystalline graphite oxide, demonstrates a high specific area and energy density [55].

**Figure 4:**
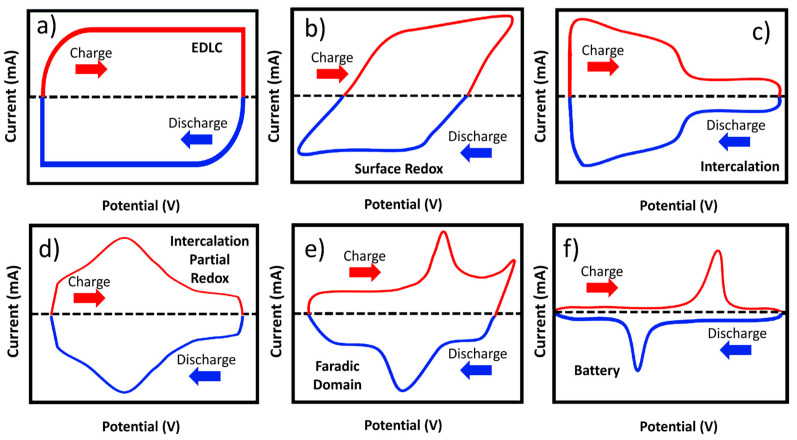
Cyclic voltammetry curves (**a**–**f**) for electrode materials based on behaviors described in the literature [50,55].

Cyclic voltammetry and galvanostatic charge and discharge processes are two means to analyze the electrochemical processes that occur in electrodes. This classification contains the guidelines that all researchers use to interpret the electrochemical results of new materials [49,56,57]. Cyclic voltammetry (CV) is the recommended tool to analyze electrochemical properties in a three-electrode configuration. For instance, EDLC electrodes present cyclic voltammetry in a square shape as shown in Figure 4a. The pseudocapacitor electrode exhibits two distinct CV shapes, which can be nearly rectangular in shape (capacitive Faradaic processes), and non-capacitive Faradaic processes (Nernstian behavior) resulting from surface redox [48,58] (see Figure 4b–d). Electrodes that exhibit battery-like behavior, so-called ’battery-grade materials’ (BGM), have been noted for presenting remarkable specific power parameters [59,60,61]. These devices’ parameters are significantly superior compared to batteries and conventional supercapacitors, presenting higher specific capacitance (Figure 4e). As the rapid phenom redox reactions modify the electrodes’ surfaces, these materials will not display rectangular cyclic voltammetry graphs (Figure 4f).

**Figure 5:**
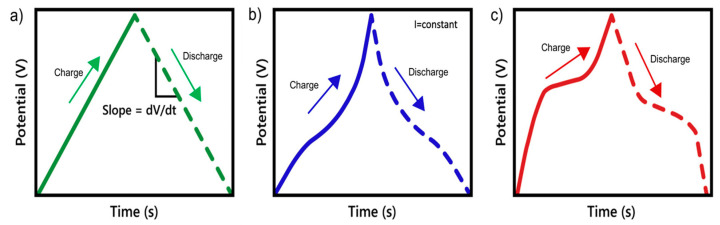
Galvanostatic charge–discharge curves for electrode materials based on charge mechanisms described in the literature [50,55], presenting the behavior of (**a**) EDLC, (**b**) pseudocapacitors, and (**c**) battery-type materials.

Another widely used method to characterize supercapacitor electrodes is the galvanostatic charge–discharge (GCD) method (Figure 5), which determines the main power of the electrode. To obtain a GCD curve, the current (i) is fixed as a function of a uniform voltage variation. For pseudocapacitor electrodes, the galvanostatic charge–discharge curve has a non-symmetrical slope (Figure 5b), contrary to the EDLC electrode, where a triangular shape is evident (Figure 5a). In the case of battery electrodes, there will be regions that have a plateau characterized by phase transformations, as demonstrated in their curve (Figure 5c) [62,63].

## 4. Influence of the Structural Properties of Electrodes

The inner surface of the electrode exhibits several properties that change to provide it a large boost in specific capacitance and charge storage. These include the pore size of the nanometer order, the interactions between ions, and the charge characteristics. The performance of a supercapacitor device strongly depends on the structure/morphology of the electrode materials; therefore, any changes in the surface morphology greatly influence its electrochemical performance. In this context, properties such as surface area, dimensions, particle architecture, solvents, controlled porosity, and stable nanocrystalline phases are essential [64,65].

An active surface through increased surface area and pore volume (mesoporous structures with a diameter of 2 nm to 50 nm) makes it easier for electrolytic ions to move and diffuse during the charge and discharge processes [65,66,67]. Thus, the morphology becomes a very important parameter in the electrochemical performance of nanomaterials for chemical electrodes. Electrodes based on Co_3_O_4_ showed great efficiency in terms of electrochemical properties. Nanostructured materials such as nanoparticles [68,69] nanowires [70], thin films [71,72], nanorods [73,74], nanoflakes [75,76], nanofibers [77,78], and hierarchical nanostructures [79] are some examples of Co_3_O_4_-based nanostructures. Further, 1D nanostructures have been considered to be a great alternative for the production of electrodes as they have high surface area, high porosity, strong electronic conductivity, and, moreover, a high production rate [80,81], thus providing more effective ion transport pathways. Thus, the morphological aspects combined with the synthesis process directly influence the chemical/physical properties of Co_3_O_4_-based materials. In Table 1 (see Section 4.5), the performance values of cobalt-oxide-based electrodes used as supercapacitors are summarized.

### 4.1. Influence of Surface Area

**Figure 6:**
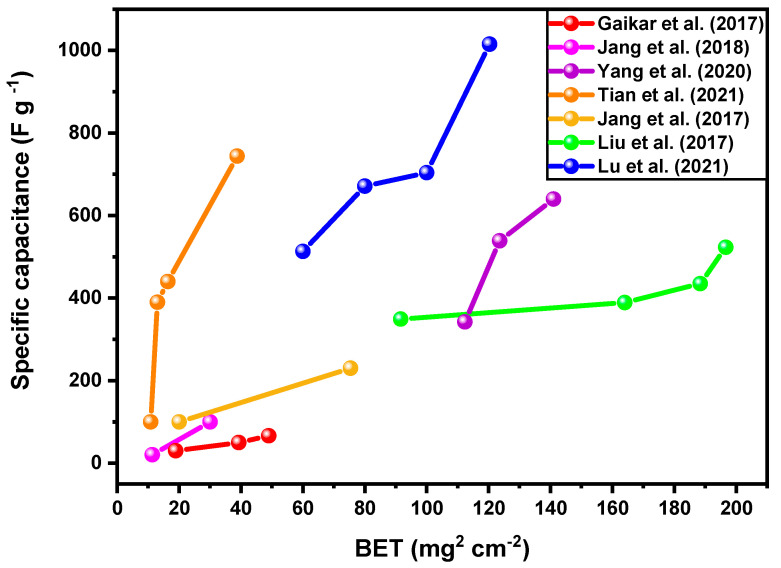
Influence of surface area based on different syntheses and morphologies on specific capacitance values for electrodes from Co_3_O_4_ materials [82,83,84,85,86,87,88].

The performance of supercapacitor electrodes in morphological terms is mainly due to the achievement of porous structures with a homogeneous distribution of pores and large surface areas, which facilitate charge transfer and result in a higher incidence of redox-active sites along the surface of the material. So, the specific capacitance tends to rise as the specific surface area (see Figure 6) and the number of pores in the structure rise. This is because pores make it easier for molecules to move through the active surface, which improves the electrochemical properties of Co_3_O_4_-based electrodes.

The characteristics of the electrodes of Co_3_O_4_ prepared by wet chemical bath deposition (CBD) using different precursor solutions such as acetate (CA), chloride (CC), and nitrate (CN) were studied by Gaikar and colleagues [82]. These samples exhibit different morphologies when calcined in an air atmosphere at 400 °C. The specific areas obtained were 18.89 m^2^ g^−1^ (CA), 39.25 m^2^ g^−1^ (CC), and 48.92 m^2^ g^−1^ (CN), and the pore volumes were 0.192, 0.190, and 0.304 cm^3^ g^−1^, respectively. This confirms the presence of mesoporous structures. Consequently, the Co_3_O_4_-CN electrode (66.40 F g^−1^) improves its charge storage capacity and electrochemical performance.

Porous electrodes of Co_3_O_4_ were prepared by hydrothermal synthesis at 120 °C using a bio-matrix of sorghum straw [83] and then calcined at 500 °C. The surface areas (26.5 m^2^ g^−1^) of the porous nanotube shapes were higher than those of the non-porous Co_3_O_4_ (11.3 m^2^ g^−1^), respectively. According to the specific capabilities, they have been estimated at 100.6 F g^−1^ (porous) and 20.5 F g^−1^ (no porosity) at a current density of 2 A g^−1^ and 90% retention after 9000 cycles.

In the work of Yang et al. [84], the Co_3_O_4_ electrode was produced by hydrothermal synthesis, presenting nanostructures with different morphologies (1D nanowires, 2D nanosheets, 3D core–shell, and hierarchical structure) in the following order according to the surface area and pore volume: nanowires (140.96 m^2^ g^−1^ and 0.41 cm^3^ g^−1^) > nanosheets (123.56 m^2^ g^−1^ and 0.25 cm^3^ g^−1^) > core–shell (112.36 m^2^ g^−1^ and 0.20 cm^3^ g^−1^) > hierarchical structure (88.58 m^2^ g^−1^ and 0.19 cm^3^ g^−1^), with all morphologies presenting mesoporous structures. The specific capacitance, calculated from the CV curves at a scan rate of 2.0 mV s^−1^, provided the highest value for nanowires (639.8 F g^−1^).

Co_3_O_4_ nanostructures were synthesized from different ion precursors and subsequently deposited on the surface of the nickel foam by the chemical bath deposition (CBD) technique [85]. The morphologies of the Co_3_O_4_ electrodes varied depending on the precursor anions. Surface area values of the Co_3_O_4_ nanostructures with different ion precursors have been investigated by BET, showing interesting differences. For instance, acetate (A-Co_3_O_4_), nitrate (N-Co_3_O_4_), chloride (C-Co_3_O_4_), and sulfide (S-Co_3_O_4_) have, respectively, surface areas of 38.73 m^2^ g^−1^ and 0.082 cm^3^ g^−1^, 10.80 m^2^ g^−1^ and 0.03 cm^3^ g^−1^, 11.00 m^2^ g^−1^ and 0.042 cm^3^ g^−1^, and 16.37 m^2^ g^−1^ and 0.044 cm^3^ g^−1^. Additionally, after 1000 charge–discharge cycles at 5 A g^−1^ in the 2M KOH electrolyte, the A-Co_3_O_4_ electrode displayed the highest specific capacitance with 743.8 F g^−1^ and good cyclic stability with 95.8% retention.

Controllable fabrication of Co/Co_3_O_4_-3D nanoporous electrodes increases the specific capacitance (902.3 F g^−1^ in 2 A g^−1^) and improves the retention cycle (78.7% at 10 A g^−1^) [89]. This resulted in obtaining a nanostructure with hierarchical coordination and pore size distribution of bimodal nanopores (4.3 and 18.2 nm) with a specific surface area of 92.60 m^2^ g^−1^, which provided electroactive sites for effective oxidation and reduction.

In general, the improved electrochemical properties of these electrodes are characterized by their large surface areas, high pore volumes, and nanometer or micrometer particles.

### 4.2. Influence of Surface Roughness

Surface roughness holds significant importance as it directly influences interactions with the surrounding environment. Higher surface roughness leads to more active sites and smaller particle sizes, thereby increasing the active surface area. As a result, the surface roughness of the electrodes generates capacitive responses as the surface roughness increases.

**Figure 7:**
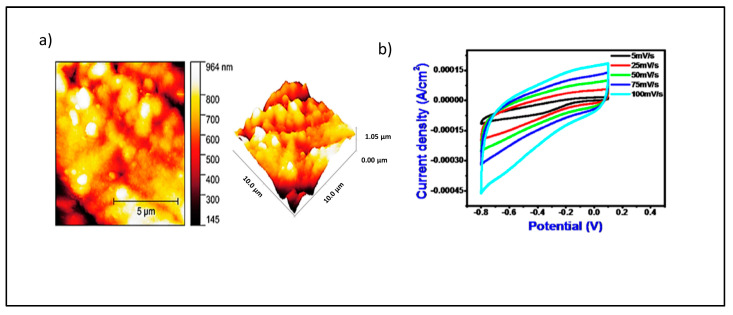
(**a**) 2D AFM and 3D AFM of Co_3_O_4_ thin-film electrode; (**b**) specific capacitance and interface capacitance versus current density graph of Co_3_O_4_ electrode (reprinted from Ref. [90], Copyright 2021, with permission of Creative Commons).

Effects of roughness on materials were also studied by Gavande et al. [90] when depositing electrolytic cobalt hydroxide on a stainless-steel substrate to produce rough thin films. The surface roughness analysis under an atomic force microscope (AFM) revealed that, as it increased, the specific capacitance of the Co_3_O_4_ electrode also increased. The thin-film Co_3_O_4_ has a specific capacitance of 284.4 F g^−1^ at a scan rate of 5 mV s^−1^ in 1M of Na_2_OH_4_, an aqueous electrolyte. In this context, the degree of surface roughness of the electrode materials is a very important factor that can evaluate the true electrochemical catalysis properties of the oxide [91]. One way to quantify electrochemical roughness of the Co_3_O_4_ electrode surface is through the cyclic voltammetry curve (Figure 7b), presenting a potential window with non-Faradaic behavior.

### 4.3. Influence of Calcination Temperature

Calcination temperature is an important parameter in the synthesis of materials for supercapacitor electrodes based on Co_3_O_4_ because it causes significant changes in the morphology of the electrodes and physical properties. Researchers generally observed that calcination temperatures within the range of 300 °C to 700 °C alter the electrolytic capacity of the electrode’s Co_3_O_4_ base. Temperature increases above 550 °C primarily affect the surface area and porosity of the material. An increase in temperature implies an increase in particle size, substantially altering the morphology and anisotropy of the materials. Changes in anisotropic physical properties decrease the supercapacitive performance of the electrodes by affecting ion diffusion kinetics, which decreases their supercapacitive performance [92].

**Figure 8:**
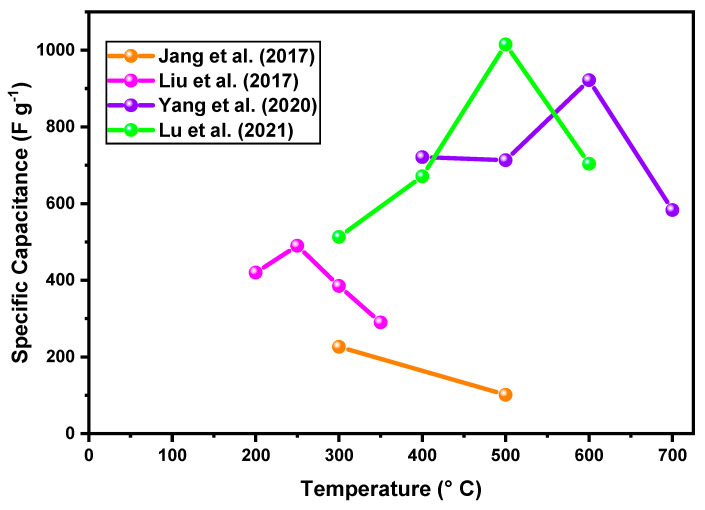
Relationship between calcination temperature and specific capacitance values for different synthesis methods in the production of Co_3_O_4_ electrodes [86,87,88,93].

Figure 8 compares the specific capacitance with the choice in the optimal electrode calcination temperature, demonstrating that nanomaterials with high surface area present capacitance optimization, even at higher temperatures. This value is based on results reported in the literature for Co_3_O_4_ electrodes obtained with different synthesis methods.

Jang et al. [86] synthesized porous cobalt oxide electrodes (NRs) via a hydrothermal method for use as supercapacitors. The application of different calcination temperatures (300 and 500 °C) provided the formation of distinct morphologies, nanorods, and nanoparticles, respectively. Increasing the temperature from 300 °C to 500 °C revealed decreases in surface area (75.36 m^2^ g^−1^ to 21.39 m^2^ g^−1^), volume of pores (0.324 cm^3^ g^−1^ to 0.132 cm^3^ g^−1^), and pore size growth (17.2 nm to 24.7 nm). This change in morphology, because of a higher calcination temperature employed, severely affected the porous surface, decreasing the transport of ions along the surface, and consequently resulted in a decrease in the capacitance value from 226.3 to 101.3 F g^−1^.

Liu et al. [87] used solvothermal precipitation synthesis at temperatures of 200, 250, 300, and 350 °C to create ultrafine cobalt oxide nanoparticles for supercapacitor electrodes. These particles had the shape of polycrystalline nanoflakes. The nanostructure had a rough, randomly arranged surface, which aided in the formation of porous structures and favored the transport of ions along short paths on the electrolyte’s surface. Analysis of the specific surface area (BET) of Co_3_O_4_-calcined electrodes at 200, 250, 300, and 350 °C demonstrated values of 188.4 m^2^ g^−1^, 196.6 m^2^ g^−1^, 164 m^2^ g^−1^, and 91.6 m^2^ g^−1^, respectively. Moreover, the pore volume (BJH) values were 0.166 cm^3^ g^−1^, 0.195 cm^3^ g^−1^, 0.448 cm^3^ g^−1^, and 0.262 cm^3^ g^−1^, respectively. For samples heated to 200 °C, 250 °C, 300 °C, and 350 °C, cyclic voltage analysis showed specific capacitance values of 435, 523, 389, and 349 F g^−1^ at a scan rate of 0.5 A g^−1^. The calcination temperature did not have a linear effect on the pore volume. However, the parameters of specific surface area and the homogeneous distribution of pore volume were decisive for achieving the best electrochemical performance.

Yang et al. 2020 [93] studied Co_3_O_4_ electrodes composed of the activity material of nanoparticles with hollow microsphere morphology and calcined at temperatures of 400–700 °C. The calcination temperature had a strong influence on the morphology of the hollow microspheres; as a result, the particles grew with the increase in the calcination temperature. The specific capacitance of electrode Co_3_O_4_-600 (922.7 F g^−1^) was higher than that of electrodes Co_3_O_4_-400 (721.0 F g^−1^), Co_3_O_4_-500 (713.5 F g^−1^), and Co_3_O_4_-700 (583.5 F g^−1^) at 1 A g^−1^, respectively. When the calcination temperature reached 700 °C, the specific capacitance decreased. This might be because the change in shape was not appropriate for permitting the solution and exposing the active sites of the redox reaction compared to electrodes that had been heated to 400, 500, and 600 °C.

Lu et al. 2021 [88] manufactured active materials based on nanoporous Co_3_O_4_ via a solvothermal route followed by calcination treatment (300 °C, 400 °C, 500 °C, and 600 °C). In an attempt to preserve the initial structure after the calcination process, Co_3_O_4_ was supported on a ZIF-67 (zeolitic) structure; however, with increasing temperature, changes in morphology occurred. The increase in calcination temperature to 500 °C was beneficial, showing a very high specific capacitance of 1015 F g^−1^ under current density of 1 A g^−1^.

In summary, it is noted that there is a level of calcination temperature where structural and morphological changes begin to occur, causing a decrease in the surface area of the active material, influencing losses in electrochemical performance above this level.

### 4.4. Influence of Surfactants

Surfactants help to control the reaction because they naturally have a number of functional groups that lower net surface tension. This prevents particles from sticking together and maintains the particle shapes. The use of surfactants significantly influences the surface morphology and physicochemical characteristics of cobalt oxide samples, with some believing that the ability to control the size of nanoparticles is the main benefit of using surfactants over other techniques [94,95].

Surfactants are classified as cationic, anionic, non-ionic, and zwitterionic (amphoteric) and can be hydrophilic and/or hydrophobic. The specific surface area and morphological characteristics of Co_3_O_4_ have an impact on the structure of the surfactant, which in turn has an impact on the electrode’s charge storage results [96,97,98].

**Figure 9:**
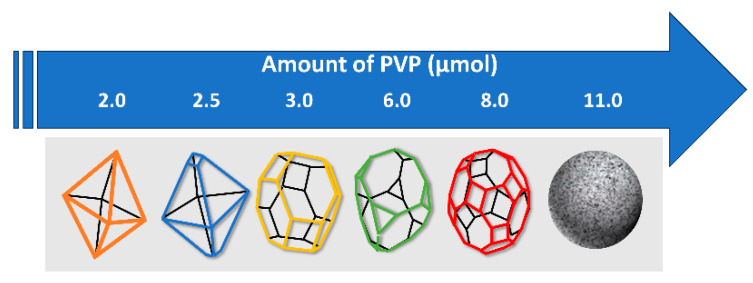
The evolution of the form of PVP-surfactant-containing Co_3_O_4_ nanoparticles based on [100].

Polyvinylpyrrolidone (PVP) is a surfactant that is often used in the synthesis of Co_3_O_4_ electrodes. With its hydroxyl groups, it dissolves in water and is a good example for creating nanomaterials. These groups help to maintain stability in materials by forming complexes or keeping ions inside the polymer system. Furthermore, adding PVP to the nanostructure synthesis will improve the size distribution of the nanocrystals and reduce nanoparticle agglomeration [99]. When the amount of PVP was increased, the morphology of the Co_3_O_4_ nanoparticles changed (Kang et al. 2016 [100]) as a consequence of the different interactions between surfactants and Co_3_O_4_ particles (see Figure 9). Interestingly, surfactants that have groups with lone pairs generate adsorption processes on the surface of the particles.

In the literature, other surfactants have also been studied/evaluated in the preparation of cobalt-oxide-based electrodes. Zhang et al. [101] tested the influence of different surfactant structures, polyvinyl-pyrrolidone-PVP, cetyltrimethylammonium bromide-CTAB, and sodium dodecyl sulphate-SDS through a hydrothermal synthesis process of Co_3_O_4_ supported by Ni foam. The results revealed that the different surfactants influenced the electrochemical characteristics of the cobalt oxide electrodes. The addition of CTAB produced scattered nanowires on Ni foam with a surface area of 92.9 m^2^ g^−1^, while the addition of SDS surfactants obtained a specific surface area of 121.4 m^2^ g^−1^. The PVP used as a precursor presented nanowires with 65.0 m^2^ g^−1^ of surface area. In the case of Co_3_O_4_-SDS, a higher specific capacitance (1217.4 F g^−1^) was evidenced after 2500 cycles.

**Figure 10:**
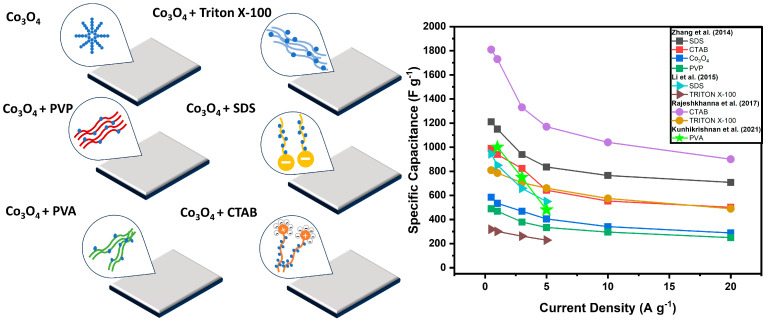
Schematic illustration of mechanism formation of morphologies of nanostructured cobalt oxide material by using different surfactants, and the specific capacitance values of the Co_3_O_4_ electrodes at different current densities [101,102,103,104].

Hydrothermal synthesis of Co_3_O_4_/graphene composites using different surfactants, SDS (sodium dodecylbenzene sulfonate) and Triton X-100 (4-octylphenol polyethoxylate), was followed by calcination of the precursors at various temperatures (250–350 °C), which exposed an ellipsoidal morphology [102]. The crystallite size of the Co_3_O_4_ composites manufactured with the addition of SDS surfactant (12.7 and 17.2 nm) was smaller than that of the Co_3_O_4_ composites prepared with Triton X-100 (15.3 and 24.1 nm) at the analyzed temperatures. After 1000 cycles, the electrodes calcined at 250 and 350 °C with the presence of SDS exhibited capacitances of 467.9 and 513.2 F g^−1^ and retentions of 95.5% and 81%, respectively. The electrode prepared with the addition of the surfactant Triton-X100 and calcined at 250 °C showed a specific capacitance of 238.5 F g ^−1^ and 97.8% retention after 1000 cycles (see Figure 10).

Rajeshkhanna et al. [103] prepared samples of cobalt oxide in surfactant-free Ni foam, with cationic surfactants (CTAB) and non-ionic surfactants (Triton X-100), synthesized via hydrothermal method. The surface areas obtained by the 66 m^2^ g^−1^ cobalt oxide electrodes (Co_3_O_4_-C) and 80 m^2^ g^−1^ (Co_3_O_4_-T) indicated that the materials contained mesopores according to the isotherms presented. The specific capacitance values of the Co_3_O_4_-C (1820 F g^−1^) and Co_3_O_4_-T (806 F g^−1^) electrodes are mainly attributed to the morphologies of the nanoflakes, which have larger pore volumes, larger surface areas, and smaller crystallite sizes. The specific capacitance of the surfactant-free electrode was 288 F g^−1^.

Cobalt oxide (Co_3_O_4_) was synthesized using a hydrothermal process followed by calcination (300 °C) using PVA (polyvinyl alcohol) in high concentrations (0, 3 and 5 g) in a 10 mL solution [104]. The concentration of PVA surfactant affected both the morphology of the electrodes and the size of the Co_3_O_4_ crystallites. The 5g PVA electrode exhibited a specific capacitance of 954.0 F g^−1^ at a current density of 1 A g^−1^, maintaining 88% capacitance after 2000 cycles at 100 mV s^−1^. The morphology obtained with the variation in the concentration of the surfactant was beneficial in obtaining high specific capacitance and good stability.

### 4.5. Influence of Oxygen Vacancy Formation

In order to improve the electrochemical properties, the introduction of oxygen vacancies, or even vacancies of the metal itself, generates changes in the crystalline and electronic structures, mainly by improving the intricate characteristics of the material [105]. This practice (method) increases electrochemical activity in materials based on transition metal oxides (Ni, Co, Fe, etc.). One of the advantages is that vacancies act as both active sites and/or electron carriers, stimulating the processes of charge and discharge during reversible redox reactions. Another advantage is that vacancies alter the structure of the active electrodes, maintaining stability, and also generate an electric field at the atomic level, improving electrochemical performance [106,107,108,109].

Some studies in the literature that focused on the production of electrodes for electrochemical supercapacitors based on Co_3_O_4_ are discussed in terms of the application of the methodology of introducing oxygen/metal vacancies in the structure of the material. The analyses of oxygen vacancies, number of carbons, and cobalt species are related to high-resolution spectra obtained via photoelectron spectroscopy (XPS). In this case, the presence of oxygen vacancies contributes to improving electrochemical conductivity in an alkaline medium [110]. When the Co^2+^/Co^3+^ ratio obtained is higher than the theoretical molar ratio of stoichiometric Co_3_O_4_, it indicates that oxygen vacancies have been formed in the structure. Therefore, by adjusting/optimizing the electronic structure of materials based on metal oxides, we increase the conductivity by introducing vacancies and consequently significantly increase the specific capacitance.

**Figure 11:**
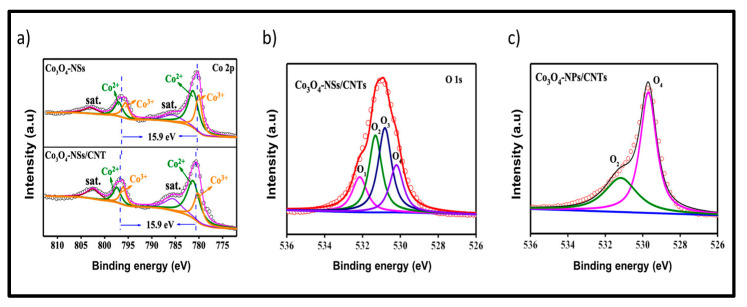
(**a**) Co_2p_ XPS spectra of Co_3_O_4_-NSs/CNTs-5% nanocomposites and Co_3_O_4_-NSs, (**b**) The red line is the XPS spectrum of O1s that was deconvoluted for the Co_3_O_4_-NSs/CNTs-5% nanocomposites, and (**c**) O_1s_ XPS spectrum of Co_3_O_4_-NPs/CNTs-5% nanocomposites (reprinted from Ref. [113], Copyright 2021, with permission from ACS Publications).

**Figure 12:**
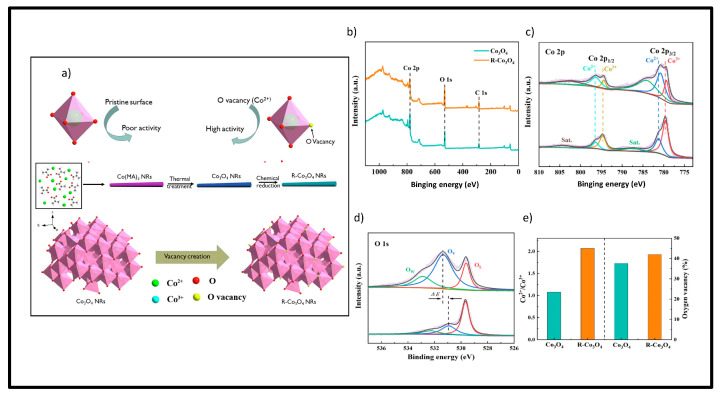
(**a**) Co_3_O_4_ nanoribbons with abundant oxygen vacancies improve electrode performance in energy storage. (**b**) Photon electron spectrometry of cobalt species. (**c**) Co 2p, (**d**) O1s (hydroxyl oxygen, oxygen vacancies and oxygen lattice, respectively) and), (**e**) The ratio of Co^2+^/Co^3+^ and the content of oxygen vacancies. (reprinted from Ref. [114], Copyright 2022, with permission from Elsevier).

**Figure 13:**
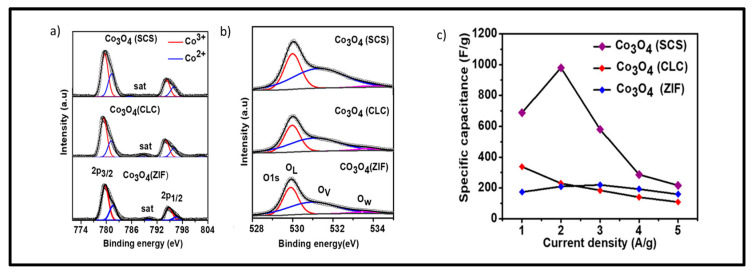
XPS data collected: (**a**) cobalt species (Co_2p_), (**b**) oxygen vacancies (O_1s_), and (**c**) specific capacitances (reprinted from Ref. [116], Copyright 2023, with permission from ACS Publications).

Several techniques can be implemented in the formation of oxygen vacancies, such as solid-state redox reactions, which use reductions from solid (NaH, LiH, CaH_2_, and graphene) or gaseous (H_2_, NH_3_, and S) reducers at high temperatures; or by a wet chemical pathway, which uses NaBH_4_ as a reducing agent at room temperature or by hydrothermal process. The literature highlights that the chemical reduction process modifies the Co^2+^/Co^3+^ ratio, and, in this case, the presence of reduced species such as CoO would increase the capacitance of the reduced electrode of Co_3_O_4_ [111,112] according to the XPS analyses presented in Figure 11, Figure 12 and Figure 13.

In the work by Zhang et al. [113], the addition of NaBH_4_ promoted the formation of ultrafine Co_3_O_4_-NSs with the presence of oxygen vacancies, acting as active sites. The ultrafine 2D morphology provided abundant active sites by shortening the electron/ion diffusion distance. The nanocomposites of Co_3_O_4_-NSs showed a mesoporous structure with a surface area of 150 m^2^ g^−1^, which was higher than that of Co_3_O_4_-NSs with the presence of CNTs-5% (135 m^2^ g^−1^,). In addition, the addition of 3% CNTs and 10% CNTs indicated specific surface areas of 145 m^2^ g^−1^ and 138 m^2^ g^−1^, respectively, showing that the introduction of carbon nanotubes had little influence on the electrode surface according to the XPS analyses (Figure 11). From the GCD analysis, the specific capacitances of Co_3_O_4_-NSs are 738.7 F g^−1^ without the presence of CNTs, 816.5 F g^−1^ (CNTs-3%), 1280.4 F g^−1^ (CNTs-5%), and 860.4 F g^−1^ (CNTs-10%), respectively. Co_3_O_4_ nanoparticles with CNTs-5% were also analyzed, with surface areas of 50 m^2^ g^−1^ and 595.4 F g^−1^ at 1 A g^−1^.

In the article by Ma et al. [114], Co_3_O_4_ nanoribbons rich in oxygen vacancy were prepared via a reduction process with NaBH_4_ prior to calcination. The achieved morphology of R-Co_3_O_4_ NRs potentiated the presence of electroactive sites, making them candidates for supercapacitor electrodes (Figure 13). The R-Co_3_O_4_ NRs obtained a specific capacitance of 465 F g^−1^, which was higher than that of the Co_3_O_4_ NRs (347.4 F g^−1^) at 1 A g^−1^.

Li et al. [115] produced cobalt oxide nanosheets grown on nickel foam (D-Co_3_O_4_@Ni) via wet reduction in NaBH_4_. The specific capacitance values of the pure and reduced electrodes were analyzed at various current densities. At a current density of 1 A g^−1^, the pure Co_3_O_4_ electrode showed 202 F g^−1^, while the reduced Co_3_O_4_ electrode showed a significantly improved specific capacitance of 963.95 F g^−1^. Finally, the retention rate of the reduced electrode (90%) that presented oxygen vacancies was 10% higher than that of the pure electrode (80%).

Halder et al., [116] prepared Co-based metal–organic structures via a combustion method. The specific surface areas of Co_3_O_4_ (CLC) and Co_3_O_4_ (ZIF) were 12.8 m^2^ g^−1^ and 7.4 m^2^ g^−1^, respectively, while that of Co_3_O_4_ (SCS) was 20.8 m^2^ g^−1^. The electrodes analyzed in a 0.1M KOH solution showed specific capacitances of 688.3 F g^−1^ (SCS), 338.3 F g ^−1^ (CLC), and 173.3 F g^−1^ (ZIF) at a current density of 1 A g^−1^.

In the work of Kalpana et al. [117], two syntheses were used to obtain Co_3_O_4_ powders with different morphologies. A co-precipitation method (Co_3_O_4_-C) and a hydrothermal method (Co_3_O_4_-H) were studied in terms of electrochemical properties as supercapacitor electrodes. From the study of the specific surface area, it was evidenced that the Co_3_O_4_–C sample obtained a higher specific surface area (88 m^2^ g^−1^) and pore volume (0.42 cm^3^ g^−1^) than the Co_3_O_4_ sample synthesized by the hydrothermal method (54 cm^2^ g^−1^ and 0.19 cm^3^ g^−1^), respectively, confirming its mesoporous nature. The XPS data collected revealed a 2:1 ratio of Co^3+^ to Co^2+^ in all the electrodes analyzed. The Co_3_O_4_–H electrode obtained a higher specific capacitance of 366 F g^−1^ versus 233 F g^−1^ for the Co_3_O_4_–C electrode at 0.5 A g^−1^ at a 1 M KOH. In this case, the presence of oxygen vacancies was paramount, although the electrode synthesized via the hydrothermal method had a smaller surface area.

Thus, the oxygen vacancies created or induced in the crystalline structures modified the electronic structure, a critical role in promoting a change in the adsorption of active species on the surface of the electrode, thus improving the electrochemical properties.

**Table 1:** Specific capacitances of Co_3_O_4_ electrodes on several supercapacitor types.

Morphology	Synthesis	Capacitance	Electrolyte	S_BET_	Retention	Ref.
		(F/g)		(m^2^/g)	(%)/Cycles	
nanoparticles	Sol–gel	318	2M KOH	-	98%/1000	[66]
nanoparticles	Hydrothermal	138.93	1M KOH	-	86.60%/-	[67]
nanowires	Chem. Deposition	850	1M KOH	66.33	86%/1000	[68]
Thin film	Pyrolysis spray	565	2M KOH	-	93.64%/1000	[69]
Thin film	Electrodeposition	396.67	0.1M Na_2_OH_4_	-	100.0%/1600	[70]
Thin film	Chem. Deposition	479.3	1M KOH	-	-	[71]
Nanoflakes	Electrodeposition	315	0.5M Na_2_SO_4_	-	98.30%/300	[72]
Nanofibers	Electrospun	731	1M KOH	24	-	[77]
Nanofibers	Electrospun	407	6M KOH	67	94.0%/1000	[78]
Nanoflowers	Hydrothermal	473	1M KOH	22.01	77.0%/5000	[79]
Thin film	Chem. Deposition	66.4	1M NaOH	48.92	90.0%/1000	[82]
Nanocubes	Hydrothermal	430	6M KOH	61.5	85.0%/1000	[81]
Porous	Hydrothermal	100.6		26.5	90.0%/9000	[83]
Nanowires	Hydrothermal	639.8	3M KOH	140.9	85.9%/5000	[84]
Thin film	Chem. Deposition	743.8	2M KOH	38.73	98.8%/1000	[85]
Nanoparticles	Calcination	902.3	6M NaOH	83.63	72.70%/1200	[89]
Thin film	Electrodeposition	284.4	1M Na_2_OH_4_	-	82.0%/1000	[90]
Nanoflakes	Solvothermal	523	6M KOH	196.6	92.0%/1500	[87]
Microspheres	Solvothermal	922	2M KOH	-	105%/10,000	[93]
Nanocubes	Solvothermal	1015	2M KOH	120.3	78.2%/5000	[88]
Nanowires	Hydrothermal	1217.4	6M KOH	121.4	100.0%/2500	[101]
Nanoparticles	Hydrothermal	513.2	1M KOH	-	95.5%/1000	[102]
Nanoflakes	Hydrothermal	1820	2M KOH	66	92.0%/2000	[103]
Nanorods	Hydrothermal	954	2M KOH	-	88.0%/2000	[104]
Nanotubes	Hydrothermal	1280	1M KOH	150	-	[113]
Nanoribbons	Reduction	465	1M KOH	-	-	[114]
Nanosheets	Reduction	963	1M KOH	-	-	[115]
MOF	Combustion	688.3	0.1M KOH	20.8	-	[116]
Nanopowder	Co-precipitation	366	1M KOH	88	-	[117]

## 5. Conclusions and Future Perspectives

This review presented and discussed several articles on cobalt-oxide-based materials as electrode candidates for electrochemical supercapacitors. In the search for the unification of the best parameters, several methods and their applications, their morphologies and structures, and their electrochemical properties were summarized. Those factors that affect electrochemical efficiency include morphology, porosity, calcination temperature, use of surfactants and precursors, the introduction of vacancies, and the choice in electrolyte.

The porous structure of dimensional materials (1D, 2D, and 3D) provides a greater number of active sites, typically accompanied by a large surface area, resulting in excellent electrical properties. In short, finding an ideal relationship between structure and electrochemical performance is fundamental to attempting to minimize errors and optimize the development of more energy-efficient and environmentally friendly storage and conversion devices.

DFT simulation and modeling are relevant to promote the study of Co_3_O_4_ materials as electrode materials for supercapacitors, aiming to guide their growth and control the morphology. This strategy can increase the energy density, cyclic stability, and rate capability, associated with an increase in electroactive sites and stronger interaction and conduction networks.

Future expectations suggest that combining nanocomposites of Co_3_O_4_-based materials could produce a new type of hybrid supercapacitor, which could improve the cycle’s stability as long as the electrochemical properties are adjusted for this purpose.
